# Omeprazole Alleviates *Aristolochia manshuriensis Kom*-Induced Acute Nephrotoxicity

**DOI:** 10.1371/journal.pone.0164215

**Published:** 2016-10-07

**Authors:** Lianmei Wang, Hongbing Zhang, Chunying Li, Yan Yi, Jing Liu, Yong Zhao, Jingzhuo Tian, Yushi Zhang, Xiaolu Wei, Yue Gao, Aihua Liang

**Affiliations:** 1 Institute of Chinese Materia Medica, China Academy of Chinese Medical Sciences, Beijing, China; 2 State Key Laboratory of Medical Molecular Biology, Department of Physiology, Institute of Basic Medical Sciences, Chinese Academy of Medical Sciences and Peking Union Medical College, Beijing, China; 3 Beijing Institute of Radiation Medicine, Beijing, China; Max Delbruck Centrum fur Molekulare Medizin Berlin Buch, GERMANY

## Abstract

*Aristolochia manshuriensis Kom* (AMK) is a member of the *Aristolochiaceae* family and is a well-known cause of aristolochic acid (AA) nephropathy. In this study, we investigated the potential of omeprazole (OM) to alleviate AMK-induced nephrotoxicity. We found that OM reduced mouse mortality caused by AMK and attenuated AMK-induced acute nephrotoxicity in rats. OM enhanced hepatic Cyp 1a1/2 and renal Cyp 1a1 expression in rats, as well as CYP 1A1 expression in human renal tubular epithelial cells (HKCs). HKCs with ectopic CYP 1A1 expression were more tolerant to AA than the control cells. Therefore, OM may alleviate AMK-mediated acute nephrotoxicity through induction of CYP 1A1. We suggest that the coadministration of OM might be beneficial for reducing of AA-induced nephrotoxicity.

## Introduction

*Aristolochia manshuriensis Kom* (AMK), a member of the *Aristolochiaceae* family, has been used as an ingredient in herbal medicines to treat arthritis, hepatitis, rheumatism, pain, tussis, obesity and snake bites for thousands of years in China, Korea and Japan [1–4]. Aristolochic acid (AA) is an active ingredient in *Aristolochia* and *Asarum* genera and is both nephrotoxic and carcinogenic [5–9]. The predominant components of AA are 8-methoxy-6-nitro-phenanthro-(3,4-d)-1,3-dioxolo-5-carboxylic acid (aristolochic acid I, AAI) and 6-nitrophenanthro-(3,4-d)-1,3-dioxolo-5-carboxylic acid (aristolochic acid II, AAII). Herbs that contain AA, such as Ma Dou Ling (*Aristolochiae fructus*), Tian Xian Teng (*Aristolochiae herba*), Qing Mu Xiang (*Radix aristolochiae*) and Xi Xin (*Asari radix et rhizoma*), are also used in traditional Chinese medicines [10]. Therefore, identifying drugs capable of detoxifying AA has clinical value.

Cytochrome P450 (CYP450) is the superfamily of enzymes that metabolize endogenous compounds and xenobiotics, including drugs, carcinogens, and environmental contaminants [11]. The CYP450 superfamily contains more than 50 isozymes, including CYP 1A1, CYP 1A2, CYP 3A1 and CYP 2E1. CYP450 enzymes are responsible for the metabolism of AA. The activation of hepatic CYP450 abrogates the metabolism of AAI [12,13]. The activity of microsomal P450 enzymes is dependent on NADPH-P450 reductase (CPR), which provides a redox partner for CYP450-catalyzed reactions [14]. Deletion of the *Cpr* gene suppresses 95% of hepatic microsomal activity [15]. Omeprazole (OM) is a proton pump inhibitor that is prescribed to treat dyspepsia, peptic ulcer disease and gastroesophageal acid reflux disorder. As OM stimulates the expression of CYP1A1/2 [16], we investigated the protective potential of OM and the underlying mechanisms of action in AMK-induced nephrotoxicity.

## Materials and Methods

### Antibodies and reagents

AMK was percolated with 95% ethanol, and 1 gram of AMK extract was concentrated from 15.87 grams of crude drug. AMK was analyzed using high-performance liquid chromatography (HPLC) on a Waters 600E system (Waters, Milford, MA, USA) equipped with ultraviolet (UV) absorbance, refractive index detectors and a C^18^ column (Kromasil, Sweden). The mobile phase was methanol-water-acetic acid (70:30:1), and the flow rate was 1.0 mL/min. Working solutions for the gavage of AMK and OM (Huayi Pharmaceutical, Yiwu, Zhejiang, China) were prepared in 0.5% sodium carboxymethylcellulose (Solarbio life science, Beijing, China). Aristolochic acid sodium salt (AA-Na) was purchased from Santa Cruz Biotechnology, Inc. (Dallas, TX, USA). CYP 1A1 antibody was purchased from BioWorld (Beijing, China). Sodium bicarbonate (SB) was purchased from Solarbio life science (Beijing, China).

### Animals

ICR mice and Sprague–Dawley rats were obtained from Vital River, a Charles River Company, Beijing, China. Mice and rats were maintained in cages in a room equipped with an air-filtering system, and they were kept on a 12-h light/dark cycle. The animals were fed standard food and given sterilized water. This study was carried out in strict accordance with the recommendations in the Guide for the Care and Use of Laboratory Animals of the Institute of Chinese Materia Medica, China Academy of Chinese Medical Sciences. The protocol was approved by the Committee on the Ethics of Animal Experiments of the Institute of Chinese Materia Medica, China Academy of Chinese Medical Sciences (Permit Number: 20142038). We used humane endpoints for animals when they were showing signs of moribund conditions including hypothermia, and 20% weight loss. Two mice reached this moribund state in the experiment, and they were euthanized. All procedures were performed while the animals were under sodium pentobarbital anesthesia, and all efforts were made to minimize suffering and to reduce the number of animals used. We provided supplemental heat and massaged the animals suffering from acute renal injury, and checked the animal health condition every day. There were no unexpected deaths during our experiments. We first anesthetized the animals and then sacrificed the animals by cervical dislocation at the endpoint of the study.

### Animal experiments

Gavage was used on 6 to 8-week-old, male ICR mice. Mice were given 5.3 g/kg AMK, 3.4 g/kg AMK, 2.2 g/kg AMK, 0.2 g/kg OM, or both 0.2 mg/kg OM and the different concentration of AMK mentioned above on 7 consecutive days (mice receiving both drugs were first given OM and then given AMK 30 minutes later). The mice were observed for 14 days since drug treatment.

SD rats underwent gavage with AMK (2.2 g/kg), OM (0.2 g/kg), SB (0.3 g/kg), both OM (0.2 mg/kg) and AMK (2.2 g/kg) (O+A), or both SB (0.3 g/kg) and AMK (2.2 g/kg) (S+A) on 7 consecutive days. In the O+A or S+A groups, the rats were first given OM or SB then given AMK 30 minutes later. The rats were sacrificed on the 8th day since treatment. Rat liver and kidney tissues were frozen at -80°C until mRNA analysis, and kidney samples were placed in 4% paraformaldehyde for histopathological evaluation. Serum and urine samples were collected in tubes and frozen at -80°C until analysis. Serum blood urea nitrogen (BUN) and creatinine (CRE) levels were measured using an enzymatic assay (Wan Tai DRD, Beijing, China). Urine neutrophil gelatinase-associated lipocalin (NGAL) was measured using an Elisa Kit (R&D systems, Emeryville, CA, USA).

SD rats underwent gavage with OM (0.2 g/kg) or SB (0.3 g/kg) for 7 consecutive days. Rats were fasted on the 6^th^ day. On the 7^th^ day, the stomach was ligated for 1 hour, 30 minutes after OM or SB administration. Then, fluid from the stomach was collected and tested using pH-indicator paper (Te zhong zhi ye gong si, Hangzhou, Zhejiang, China).

### Histopathology

Rat kidneys were fixed in 4% paraformaldehyde, embedded in paraffin, sectioned at a thickness of 3 μm, and then stained with hematoxylin and eosin (H&E) for morphological evaluation. The histological alterations observed in the slides were blindly scored, as described previously [17]. Briefly, the severity of renal tubular damage was scored by calculating the percentage of tubules in the cortex medulla junction that exhibiting necrosis, luminal necrotic debris, or tubular dilation: 0, none; 1, <5%; 2, 5 to 25%; 3, 25 to 75%; and 4, >75%. The severity of renal inflammatory interstitial infiltration was scored as follows: 0, no evidence of injury; 1, minimal injury; 2, mild injury; 3, moderate injury; and 4, severe injury. All of the evaluations were performed on ten fields per section under 200×magnification.

### Quantitative real-time PCR

RNA was extracted from kidney and liver tissues as well as human renal tubular epithelial cells (HKCs) using a Total RNA Kit (OMEGA, Norcross, GA, USA). Aliquots containing 1 μg of RNA were used for reverse transcription with an oligo-dT primer (Toyobo, Osaka, Japan). qPCR was performed as previously described [18] using a Roche 480 instrument and SYBR Green PCR Master Mix (Roche, Mannheim, Deutschland) for the genes and corresponding primers (Sangon Biotech, Beijing, China) listed in S1 Table.

### Generation of stable human renal tubular epithelial cell line expressing ectopic CYP 1A1

HKCs were obtained from Peking Union Medical College Cell Culture Center (Beijing, China). The cells were cultured in DMEM/F12 (1:1) supplemented with 10% fetal bovine serum, penicillin (100 U/ml) and streptomycin (50 μg/ml) (Thermo Fisher Scientific, Waltham, MA, USA) in an incubator with a humidified atmosphere of 5% CO_2_ and 95% air at 37°C.

The CYP 1A1 cDNA was amplified by forward (5’-TGTAAGCTTATGCTTTT CCCAATCTC-3’) and reverse (5’-ATATCTAGACTAAGAGCGCAGCTGC-3’) oligonucleotides using a cDNA template obtained from Sino Biological (Beijing, China). The PCR products were digested with HindIII and XbaI and inserted into a modified pcDNA6/V5-HisB plasmid (Thermo Fisher Scientific, Waltham, MA, USA). The plasmids were then transfected into HKCs using Lipofectamine 2000 (Thermo Fisher Scientific, Waltham, MA, USA). Stable cells were selected for 15–20 days using culture media containing 10 μg/mL blasticidin (Thermo Fisher Scientific, Waltham, MA, USA). Stable transformants were maintained in DMEM/F12 (1:1) containing 10 μg/mL blasticidin.

### Cell proliferation assay (MTT)

Cell proliferation was measured using an MTT Assay Kit (BioDev-Tech, Beijing, China). A total of 8000–10000 cells per well were seeded in triplicate in 96-well plates and cultured for 1 d; the cells were then treated with OM, AA, or both. Subsequently, the cells were incubated with 150 μL of fresh medium containing 15 μL of MTT reagent [3-(4,5-dimethyl-2-thiazolyl)-2,5- diphenyl-2H-tetrazolium bromide; 5 mg/mL in PBS] at 37°C for 1–4 h. Then, 150 μL of DMSO was added to each well. The plates were incubated on a shaking platform for 10 min in the dark. The absorbance at 490 nm was determined using a microplate reader. The viability ratio was calculated as the following: optical density (OD) value of the sample/OD value of the control × 100%.

### Immunoblotting

Whole cells were lysed in lysis buffer [2% SDS, 10% glycerol, 10 mM Tris (pH 6.8), and 100 mM DTT], boiled at 98°C for 10 min, and then subjected to immunoblotting, as previously described [19].

### Statistical analysis

The data are shown as the mean ± SEM. Comparisons between groups were performed using 2-tailed Student’s t-tests. Statistical analyses were performed using Prism 5.0 software (GraphPad Software Inc.), and P values less than 0.05 were considered significant.

## Results

### Omeprazole reduces *Aristolochia manshuriensis Kom*-induced lethality in mice

**Fig 1:**
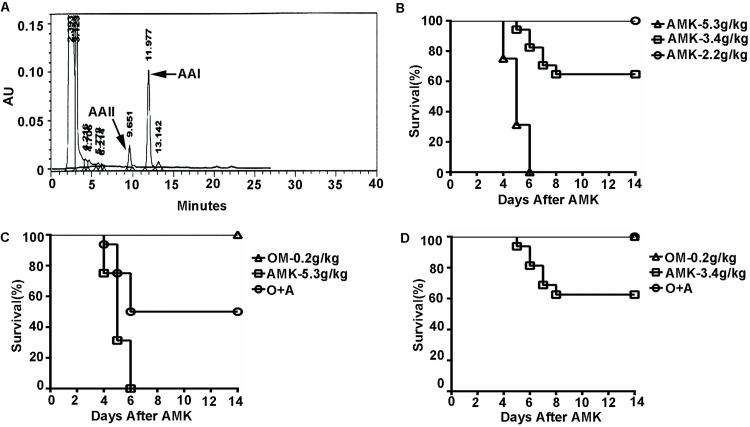
Omeprazole reduces *Aristolochia manshuriensis Kom*-induced lethality in mice. (A) HPLC analysis of AAI and AAII in AMK extracts. (B) Kaplan-Meier survival curves of mice following treatment with different doses of AMK. (C-D) Kaplan-Meier survival curves of mice indicating the protective effect of OM against lethality in mice treated with different doses of AMK, including (C) 5.3 g/kg AMK and (D) 3.4 g/kg AMK. AMK, *Aristolochia manshuriensis Kom*; OM, omeprazole; O+A, omeprazole and AMK. n = 16.

To address the protective effect of OM on AMK-mediated nephrotoxicity, we first detected 1.09% AA in the AMK extract (Fig 1A). AMK was then administered at a dose of 5.3 g/kg, 3.4 g/kg or 2.2 g/kg to mice per dose. All mice in the 5.3 g/kg group died within 6 days after AMK administration, 37.5% mice in the 3.4 g/kg group died by day 8 after AMK administration, and the remaining mice survived to the endpoint of our study, i.e., day 14. All mice in the 2.2 g/kg group survived to day 14 (Fig 1B). When the mice were pretreated with OM 30 minutes before AMK intragastric gavage, there was a significant reduction in lethality in each treatment group (Fig 1C and 1D). Specifically, the mice receiving 3.4 g/kg AMK all survived when also given OM (Fig 1D). Collectively, these results suggest that OM reduces AMK-induced lethality in mice.

### Omeprazole protects rats against *Aristolochia manshuriensis Kom*-induced renal nephrotoxicity

**Fig 2:**
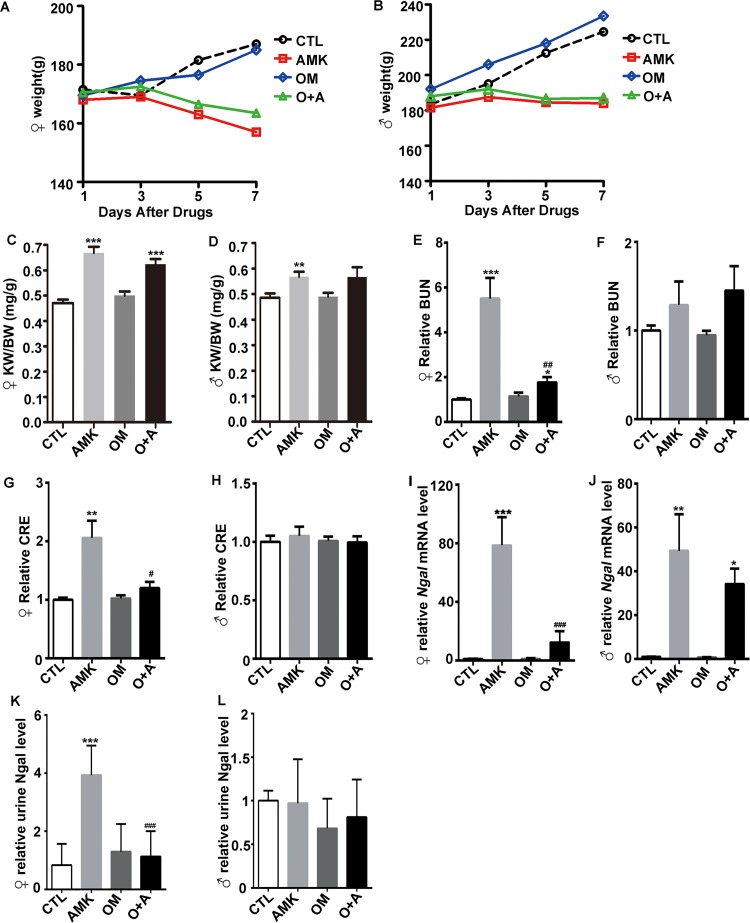
Omeprazole protects rats against *Aristolochia manshuriensis Kom*-mediated renal injury. Alterations in (A) female and (B) male body weight, (C) female and (D) male Kidney weight body weight ratio (KW/BW), (E) female and (F) male serum BUN, and (G) female and (H) male serum CRE in the four rat cohorts. qPCR analysis of (I) female and (J) male renal *ngal* mRNA levels in the four rat cohorts. Urine Ngal levels of (K) female and (L) males in each cohort. CTL, control; AMK, *Aristolochia manshuriensis Kom*; OM, omeprazole; O+A, omeprazole and AMK. Data are expressed as mean ± SEM. *P<0.05 *vs*. CTL cohort; **P<0.01 *vs*. CTL cohort; ***P<0.001 *vs*. CTL cohort; ^#^ P<0.05 *vs*. AMK cohort; ^##^ P<0.01 *vs*. AMK cohort; ^###^ P<0.001 *vs*. AMK cohort. n = 6~7.

SD rats were treated with 2.2 g/kg AMK, 0.2 g/kg OM, or a combination of OM and AMK (O+A). Following treatment, weight loss was observed in the AMK and O+A groups compared with the control (CTL) group (Fig 2A and 2B). The kidney-to-body weight ratio (KW/BW) was significantly higher in the AMK and O+A groups than in the control and OM groups in female rats (P<0.001) (Fig 2C). The KW/BW of the AMK-treated male rats was higher than that of the control cohort (Fig 2D). BUN and CRE are important indicators for clinically evaluating acute renal injury. AMK-treated female rats had dramatically higher BUN levels (P<0.001) compared with those of the control and the O+A-treated rats, which were comparable to each other (Fig 2E); serum CRE exhibited similar changes in the different female cohorts (Fig 2G). However, no obvious changes in the levels of BUN and CRE were observed in the male cohorts (Fig 2F and 2H). These results showed that female rats are more susceptible and have more severe nephrotoxicity following AMK treatment than male rats. Urine NGAL is a biomarker for renal injury. Kidney *Ngal* mRNA expression and urine Ngal levels were elevated in the AMK-treated female cohort (P<0.001) compared with the control, and these levels returned to within normal ranges upon treatment with OM (O+A group) (P<0.001) (Fig 2I and 2K). Nevertheless, kidney *Ngal* mRNA increased both in the AMK and O+A male cohort compared with the control cohort (Fig 2J). There were no obvious alterations in the level of urine NGAL in the male cohorts (Fig 2L).

**Fig 3:**
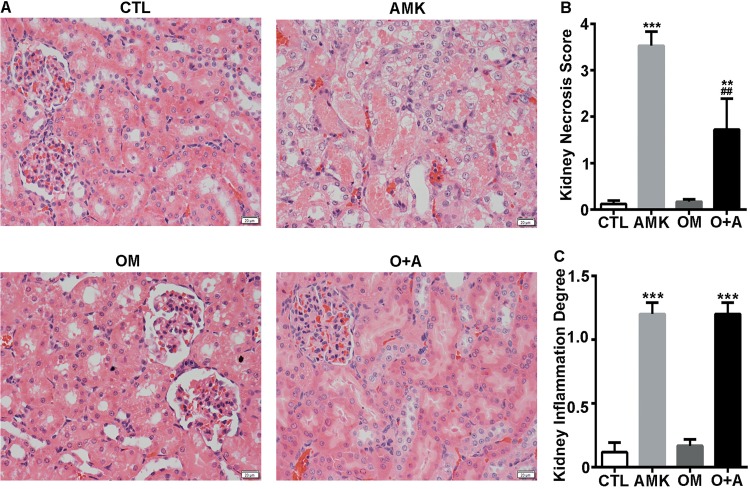
OM reverses *Aristolochia manshuriensis Kom*-induced renal damage in female rats. (A) H&E staining of rat kidney tissue from the CTL, AMK, OM, and O+A cohorts. (B) Histological damage score and (C) inflammation degree of the kidney in four cohorts. CTL, control; AMK, *Aristolochia manshuriensis Kom*; OM, omeprazole; O+A, omeprazole and AMK. Data are expressed as mean ± SEM. **P<0.01 *vs*. CTL cohort; ***P<0.001 *vs*. CTL cohort; ^##^ P<0.01 *vs*. AMK cohort. n = 6.

We analyzed the kidneys in each cohort to assess kidney injury morphologically. While tubular necrosis and dilation (i.e., atrophy) were observed after AMK administration in both female and male rats, it was more severe in the female rats (Fig 3 and S1 Fig). In contrast, the extent of tubular necrosis and dilation was significantly lower in the O+A-treated female rats than in the AMK-treated female rats (Fig 3). Although the serum BUN and CRE, kidney *Ngal* mRNA expression and urine NGAL levels were not overtly altered in the male O+A group compared with the AMK group, we found that the extent of kidney tubular necrosis and dilation in male rats was lower in the O+A group than in the AMK group (S1 Fig). Slight inflammatory interstitial infiltration was observed both in AMK and O+A cohort (Fig 3 and S1 Fig). However, no fibrosis was observed in either cohort. Taken together, these results indicate that OM pretreatment reduces AMK-induced kidney damage in rats, and that female and male rats react differently to AMK treatment (Figs 2 and 3 and S1 Fig).

**Fig 4:**
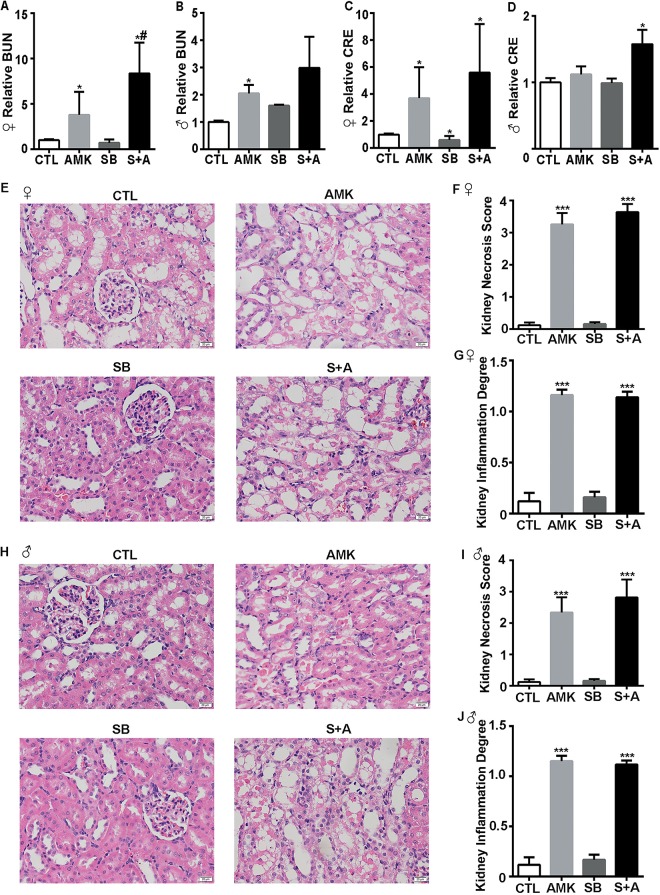
Sodium bicarbonate slightly enhances *Aristolochia manshuriensis Kom*-mediated renal nephrotoxicity. Alterations in (A) female and (B) male BUN and, (C) female and (D) male CRE levels in the four rat cohorts. (E) H&E staining of female rat kidney tissue in the four rat cohorts. (F) Histological damage score and (G) inflammation degree of the female kidney in four cohorts. (H) H&E staining of male rat kidney tissue in the four cohorts. (I) Histological damage score and (J) inflammation degree of the male kidney in four cohorts. CTL, control; AMK, *Aristolochia manshuriensis Kom*; SB, sodium bicarbonate; S+A, sodium bicarbonate and AMK. Data are expressed as mean ± SEM. *P<0.05 *vs*. CTL cohort; ***P<0.001 *vs*. CTL cohort; ^#^ P<0.05 *vs*. AMK cohort; n = 5.

At the dose of 0.2 g/kg used in this study, OM might change the stomach pH level and alter AMK absorptions. The stomach fluid from rats treating with either SB or OM, was analyzed with pH-indicator paper. SB and OM have a similar ability to increase the pH level of stomach fluid (S2 Fig). We further analyzed the effect of SB on AMK-induced renal nephrotoxicity. SD rats were treated with 2.2 g/kg AMK, 0.3 g/kg SB, or a combination of SB and AMK (S+A). The females BUN increased in S+A cohort compared with AMK cohort, however the female CRE and, male BUN and CRE levels in the S+A and AMK groups were similar (Fig 4A–4D). Morphological signs of kidney tubular necrosis and dilation slightly increased in S+A cohort compared with AMK cohort (Fig 4E, 4F, 4H and 4I). Slight inflammatory interstitial infiltration was observed both in AMK and S+A cohort (Fig 4E, 4G, 4H and 4J). However, no fibrosis was observed in either cohort. Therefore, SB slightly increased the renal nephrotoxicity mediated by AMK treatment. As a whole, these results suggest that OM might not reduce AMK-induced renal nephrotoxicity by affecting AMK absorption.

### Omeprazole antagonizes *Aristolochia manshuriensis Kom*-induced renal injury through upregulation of CYP 1A1

**Fig 5:**
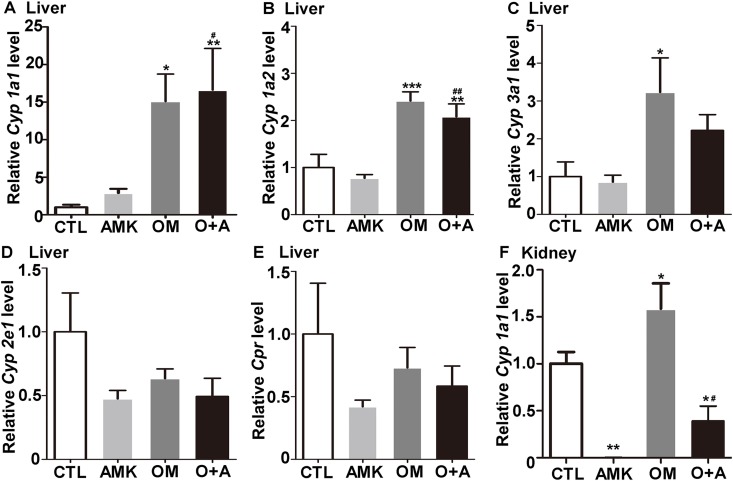
Omeprazole induces CYP450 expression in female rats. qPCR analysis of hepatic (A) *Cyp 1a1*, (B) *Cyp 1a2*, (C) *Cyp 3a1*, (D) *Cyp 2e1*, (E) *Cpr* and renal (F) *Cyp 1a1* mRNA levels in the four rat cohorts. CTL, control; AMK, *Aristolochia manshuriensis Kom*; OM, omeprazole; O+A, omeprazole and AMK. Data are expressed as mean ± SEM. *P<0.05 *vs*. CTL cohort; **P<0.01 *vs*. CTL cohort; ***P<0.001 *vs*. CTL cohort; ^#^ P<0.05 *vs*. AMK cohort; ^##^ P<0.01 *vs*. AMK cohort. n = 6.

Studies have shown that OM increases the transcription of *CYP 1A1* and *CYP 1A2* [16]. Therefore, we hypothesized that OM protects rats against AMK-induced renal injury through upregulation of CYP450. RNA was extracted from rat liver tissue to analyze the levels of *Cyp 1a1*, *Cyp 1a2*, *Cyp 3a1*, *Cyp 2e1* and *Cpr*. OM was found to drastically induced the expression of *Cyp 1a1*, *Cyp 1a2*, and *Cyp 3a1* (Fig 5A–5C and S3A–S3C Fig). However, *Cyp 2e1* and *Cpr* were not obviously altered in any cohort (Fig 5D and 5E, S3D and S3E Fig). In addition, OM induced the mRNA expression of *Cyp 1a1* in rat kidney tissue (Fig 5F and S3F Fig), while kidney *Cyp 1a2* was barely detected.

**Fig 6:**
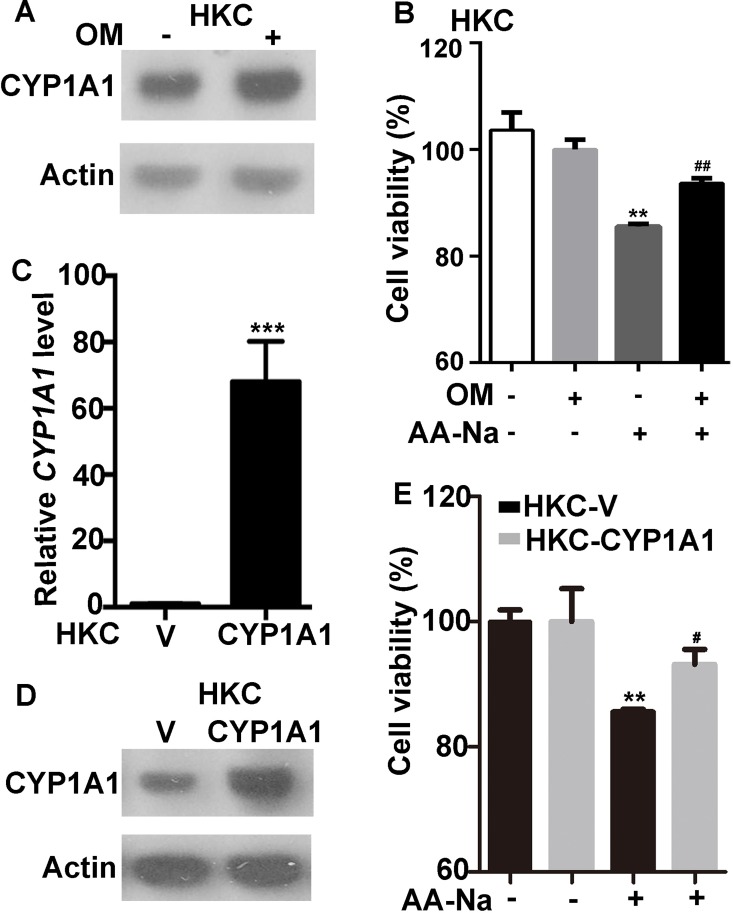
Omeprazole blocks AA-induced cell injury by upregulating CYP 1A1. (A) OM induced CYP 1A1 in HKCs. (B) HKCs cells were treated with or without 90 μM OM for 18 h before the addition of 20 μM AA-Na for 48 h. Cell viability was then detected using an MTT assay. **P≤0.01 *vs*. HKCs; ^##^ P≤0.01 *vs*. HKCs after 20 μM AA-Na administration. (C-D) HKCs were stably transfected with PCDNA6-CYP 1A1 (HKC-CYP 1A1) or PCDNA6 (HKC-V; control), and CYP 1A1 was ectopically expressed at the (C) mRNA and (D) protein levels in the targeted cell line. (E) HKC-V and HKC-CYP 1A1 cells were treated with or without 20 μM AA-Na or solvent control for 48 h. Cell viability was detected using an MTT assay. **P≤0.01 *vs*. HKC-V cells; ^#^P≤0.05 *vs*. HKC-V cells treated with AA-Na. OM, omeprazole; AA-Na, aristolochic acid sodium salt. Data are expressed as mean ± SEM.

In addition, OM (90 μM) induced the expression of CYP 1A1 in HKCs (Fig 6A). The OM pretreatment protected HKCs from AA-Na-induced (20 μM) cell death (Fig 6B). To test whether OM protects cells from AA-mediated cell injury through the upregulation of CYP 1A1, we induced the overexpression of CYP 1A1 in HKCs (HKC-CYP 1A1 cells) (Fig 6C and 6D) and evaluated the viability of these paired cell lines with or without 20 μM AA-Na. The HKC-CYP 1A1 cells were much more resilient to AA-Na than the control HKC-V cells (Fig 6E). Therefore, OM likely suppresses AA-mediated kidney injury through upregulating CYP 1A1.

## Discussion

AMK is a common cause of herbal medicine-associated nephropathy. In this study, we found that OM reduced AMK-mediated mortality in mice and attenuated AMK-induced renal injury in rats. OM increased hepatic Cyp 1a1/2 and renal Cyp 1a1 expression in rats as well as CYP 1A1 expression in HKCs. Furthermore, HKCs cells with ectopic expression of CYP 1A1 were more tolerant to AA than the control cells. Therefore, we suggest that OM suppresses AMK-induced acute nephrotoxicity likely through upregulation of CYP 1A1.

AMK causes kidney injury due to the active ingredient AA. The ingestion of AA is associated with the development of AA nephropathy (AAN) and Balkan endemic nephropathy (BEN). AMK was found to be lethal in mice in a dose-dependent manner (Fig 1B). We also observed a toxic effect of AMK in rats, as demonstrated by significantly elevated serum CRE and BUN levels in AMK-treated female rats (Fig 2E and 2G). However, no significant differences were observed in the male rats (Fig 2F and 2H). In addition, kidney lesions due to AMK treatment were more severe in female rats than in male rats (Fig 3 and S1 Fig). Therefore, we conclude that female rats are more susceptible and have more severe nephrotoxicity following AMK treatment than male rats. Our results are consistent with those reported by Mengs et al, which showed an lower median lethal dose of AA in female rats compared to the male rats [20]. Interestingly, a small cohort study in Japan found that following the ingestion of Chinese medicines containing AA, the male-to-female ratio of AAN patients was approximately 1: 1.5, indicating that AAN patients are predominately female [21]. In addition, AA-induced upper urinary tract urothelial carcinoma patients tend to be female [22]. Therefore, the results of our study are in agreement with previously published clinical data.

The biochemical analyses, including the serum concentrations of BUN and CRE, urine NGAL, and renal morphology, showed that OM decreased AMK-induced acute nephrotoxicity in female rats (Fig 2E, 2G, 2I and 2K). In contrast, the biochemical analyses indicated that OM did not alter AMK-induced acute nephrotoxicity in male rats (Fig 2F, 2H, 2J and 2L). We speculate that in the male rats, the AMK dose was too low for the biochemical analyses to detect the alleviation of AMK-induced acute nephrotoxicity by the OM pretreatment. However, we did find that OM decreased AMK-induced renal morphological alteration in male rats (S1 Fig). Therefore, we conclude that OM alleviates AMK-induced acute nephrotoxicity in both female and male rats.

AAN and BEN are characterized by chronic renal failure, tubule interstitial fibrosis and urothelial cancer [1,8,22]. AA-DNA adducts, mainly 7-(deoxyadenosine-N6-yl) aristolactam I (dA-AAI), in the kidney of AAN and BEN patients are biomarkers of exposure to AA [23]. The most abundant and persistent adduct, dA-AAI, leads to characteristic AT→TA transversions in pivotal oncogenes (e.g, the TP53 tumor suppressor gene) [24], indicating a molecular mechanism related to AA-mediated carcinogenesis. CYP450 metabolizes endogenous compounds and xenobiotics [11]. CYP 1A1 and CYP 1A2 have been reported to be involved in the metabolism of AA in mice [12]. AA is either reductively activated to form AA-DNA adducts or oxidatively detoxified to form 8-hydroxyaristolochic acid (AAIa) by CYP 1A1/2. The main role of these enzymes in vivo is to mediate the detoxification of AA and overcome the activation of AA, thereby suppressing nephrotoxicity [25]. Indeed, we found that OM induced hepatic Cyp 1a1/2 and renal Cyp 1a1 expression in rats (Fig 5 and S3 Fig). In addition, OM protected HKCs against AA-induced cell death through the upregulation of CYP 1A1 (Fig 6). Therefore, we speculate that OM might attenuate AMK-induced renal injury through enhancement of CYP 1A1 expression and further reduction of AA-DNA adducts.

Omeprazole has been reported to cause acute interstitial nephritis after an average of 2.7 months of clinical treatment with 20–40 mg per day [26]. However, in our study, OM did not cause acute nephrotoxicity (Figs 2 and 3 and S1 Fig), as demonstrated by the normal CRE and BUN serum levels and kidney morphology observed in OM-treated rats. Altered absorptions or metabolisms are two of the known, major mechanisms of drug-drug interactions. One study reported that treating mice for 7 days with an OM dose of 0.4 g/kg decreased acid secretion by 97% [27]. We found that SB and OM altered the pH level of stomach fluid in a similar manner (S2 Fig). As a vehicle for dissolving AA [28], SB slightly increased the renal nephrotoxicity mediated by AMK treatment in our work (Fig 4). It was reported previously that serum CRE levels increased in AAN patients after treated with SB [21]. Therefore, these results suggest that OM might not reduce AMK-mediated renal nephrotoxicity by affecting AMK absorption.

In this study, we found that OM protected against AMK-induced renal injury. Studies have shown that 3-methylcholanthrene (3-MC), β-naphthoflavone (BNF) and tanshinone I protect mice against AA-induced nephrotoxicity [12,29,11]. 3-MC and BNF activate the aryl hydrocarbon receptor [30], resulting in the transcriptional induction of CYP 1A1/2 expression [31–34]. Tanshinone I is extracted from the traditional Chinese medicine Salvia miltiorrhiza Bunge and stimulates CYP 1A1/2 expression [11,35]. However, both 3-MC and BNF are genotoxic and carcinogenic [36–39]. Tanshinone I is not well characterized and has not been tested for the treatment of AAN. Therefore, we explored alternative choice to alleviate AAN. OM is one of the most widely prescribed drugs internationally and has been used as a medicine for almost 30 years. OM is available over the counter in some countries, has low toxicity and is affordable for patients. We demonstrated that OM alleviated AMK-mediated renal damage in rats and improved the survival of AMK-poisoned mice. Our work demonstrates that coadministration of OM might be beneficial for reducing AA-induced nephrotoxicity.

## Conclusions

Our findings showed that OM reduced mouse mortality caused by AMK and attenuated AMK-induced acute nephrotoxicity in rats. OM was found to alleviate AMK-mediated acute nephrotoxicity through the augmentation of Cyp 1A1.

## Supporting Information

S1 Fig Omeprazole reverses *Aristolochia manshuriensis Kom*-induced kidney damage in male rats. (A) H&E staining of male rat kidney tissue samples from the CTL, AMK, OM, and O+A cohorts. (B) Histological damage score and (C) inflammation degree of the kidney in the four cohorts. CTL, control; AMK, *Aristolochia manshuriensis Kom*; OM, omeprazole; O+A, omeprazole and AMK. Data are expressed as mean ± SEM. *P<0.05 *vs*. CTL cohort; ***P<0.001 *vs*. CTL cohort; ^##^ P<0.01 *vs*. AMK cohort. n = 6.
(TIF)

S2 Fig Omeprazole and sodium bicarbonate increase the pH level of stomach fluid. (A) Schematic illustration of the pH-indicator paper. (B) The pH level of stomach fluid in the CTL, SB and OM cohorts. CTL, control; SB, sodium bicarbonate; OM, omeprazole. n = 8.
(TIF)

S3 Fig Omeprazole induces the expression of CYP450 in male rats. qPCR analysis of hepatic (A) *Cyp 1a1*, (B) *Cyp 1a2*, (C) *Cyp 3a1*, (D) *Cyp 2e1*, and (E) *Cpr* and renal (F) *Cyp 1a1* mRNA levels in the four rat cohorts. CTL, control; AMK, *Aristolochia manshuriensis Kom*; OM, omeprazole; O+A, omeprazole and AMK. Data are expressed as mean ± SEM. *P<0.05 *vs*. CTL cohort; **P<0.01 *vs*. CTL cohort; ***P<0.001 *vs*. CTL cohort; ^#^ P<0.05 *vs*. AMK cohort; ^##^ P<0.01 *vs*. AMK cohort. n = 6.
(TIF)

S1 Table qPCR primer sequences. (DOC)
