# Supplementary figures and images for: Omeprazole Alleviates Aristolochia manshuriensis Kom-Induced Acute Nephrotoxicity
Source: PLoS One. 2016 Oct 7;11(10):e0164215. doi: 10.1371/journal.pone.0164215 (PMC5055352; doi:10.1371/journal.pone.0164215)

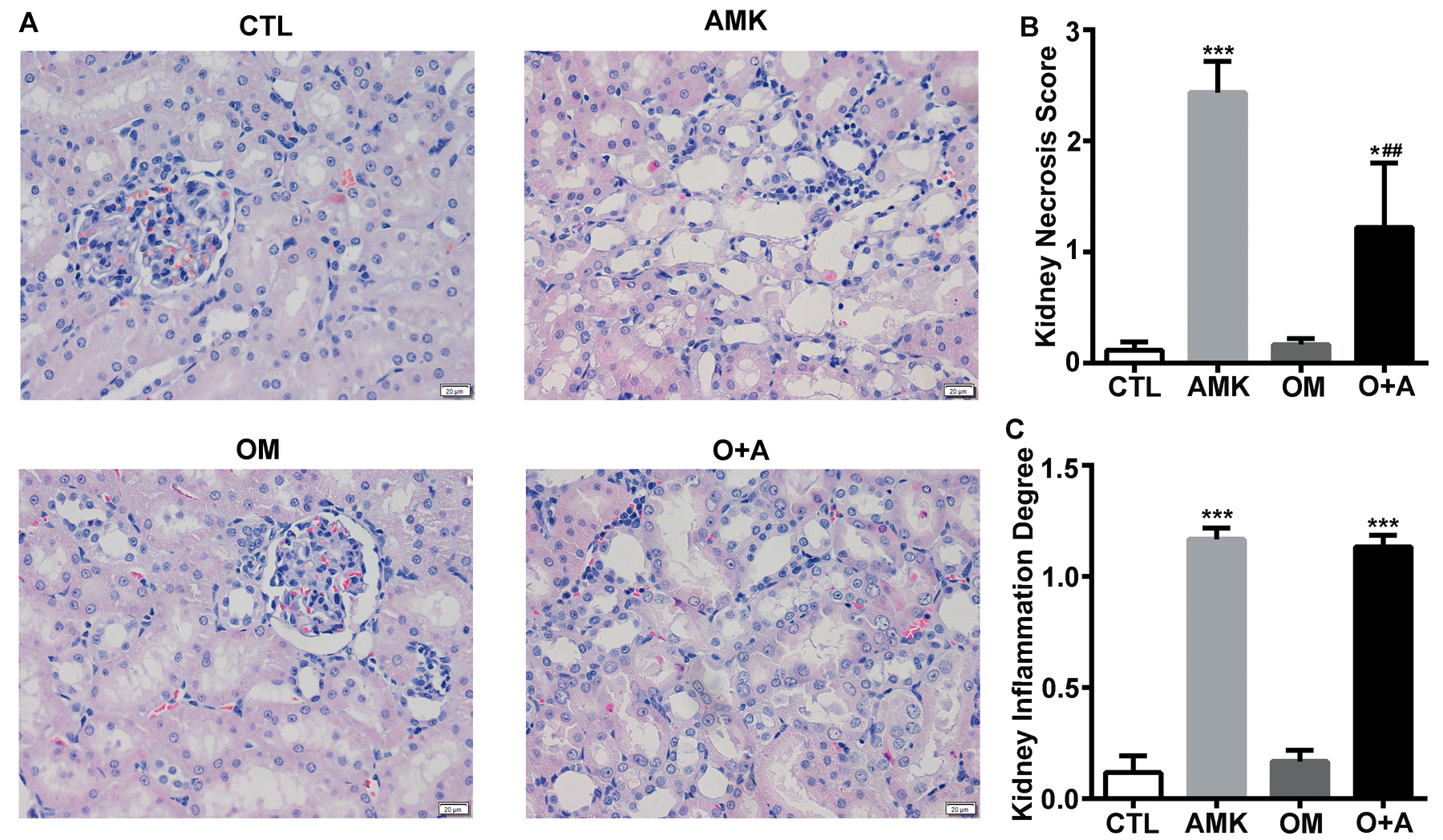

Supplement: S1 Fig — (A) H&E staining of male rat kidney tissue samples from the CTL, AMK, OM, and O+A cohorts. (B) Histological damage score and (C) inflammation degree of the kidney in the four cohorts. CTL, control; AMK, Aristolochia manshuriensis Kom; OM, omeprazole; O+A, omeprazole and AMK. Data are expressed as mean ± SEM. *P<0.05 vs. CTL cohort; ***P<0.001 vs. CTL cohort; ## P<0.01 vs. AMK cohort. n = 6. (TIF) [file pone.0164215.s001.tif]

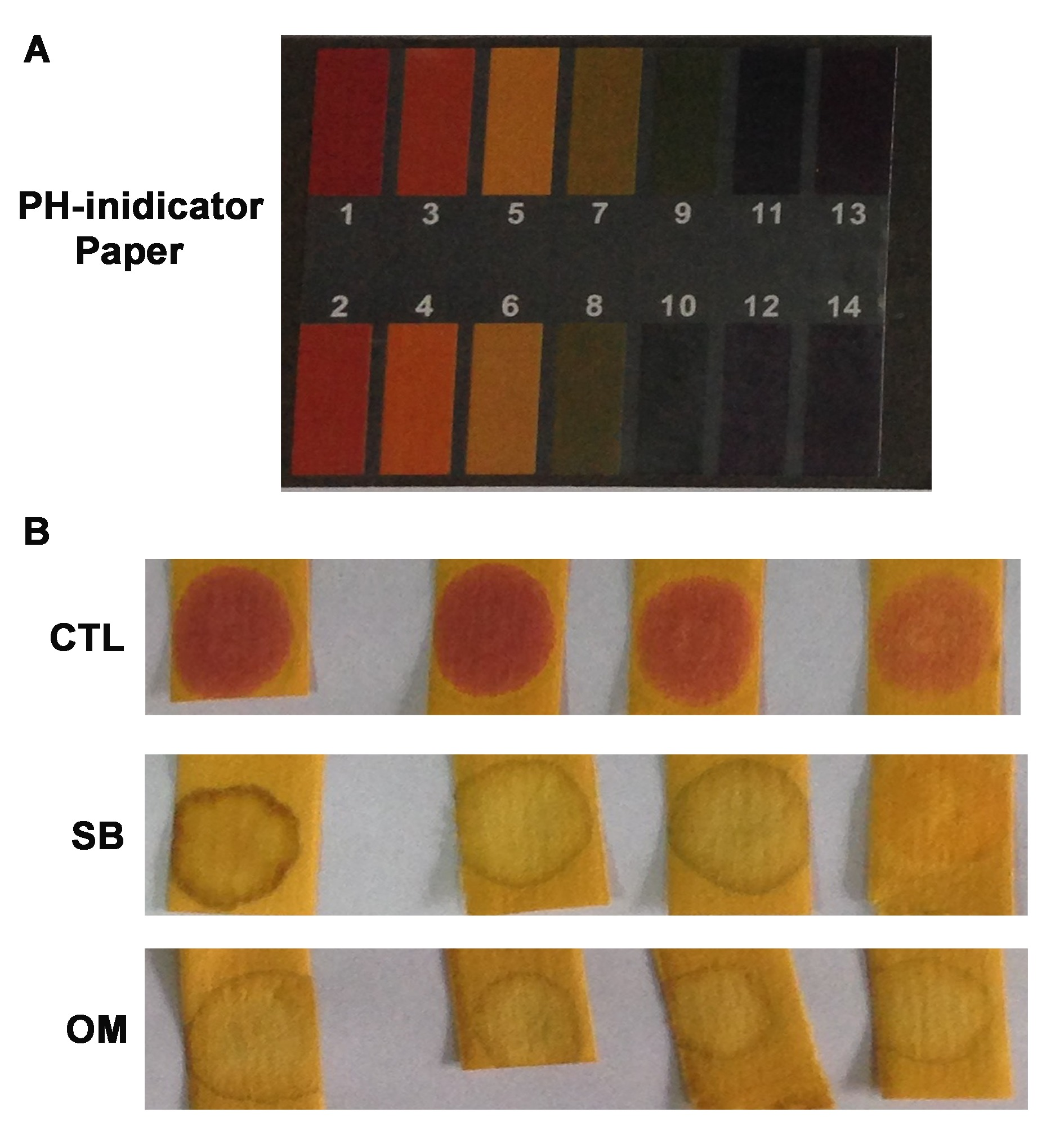

Supplement: S2 Fig — (A) Schematic illustration of the pH-indicator paper. (B) The pH level of stomach fluid in the CTL, SB and OM cohorts. CTL, control; SB, sodium bicarbonate; OM, omeprazole. n = 8. (TIF) [file pone.0164215.s002.tif]

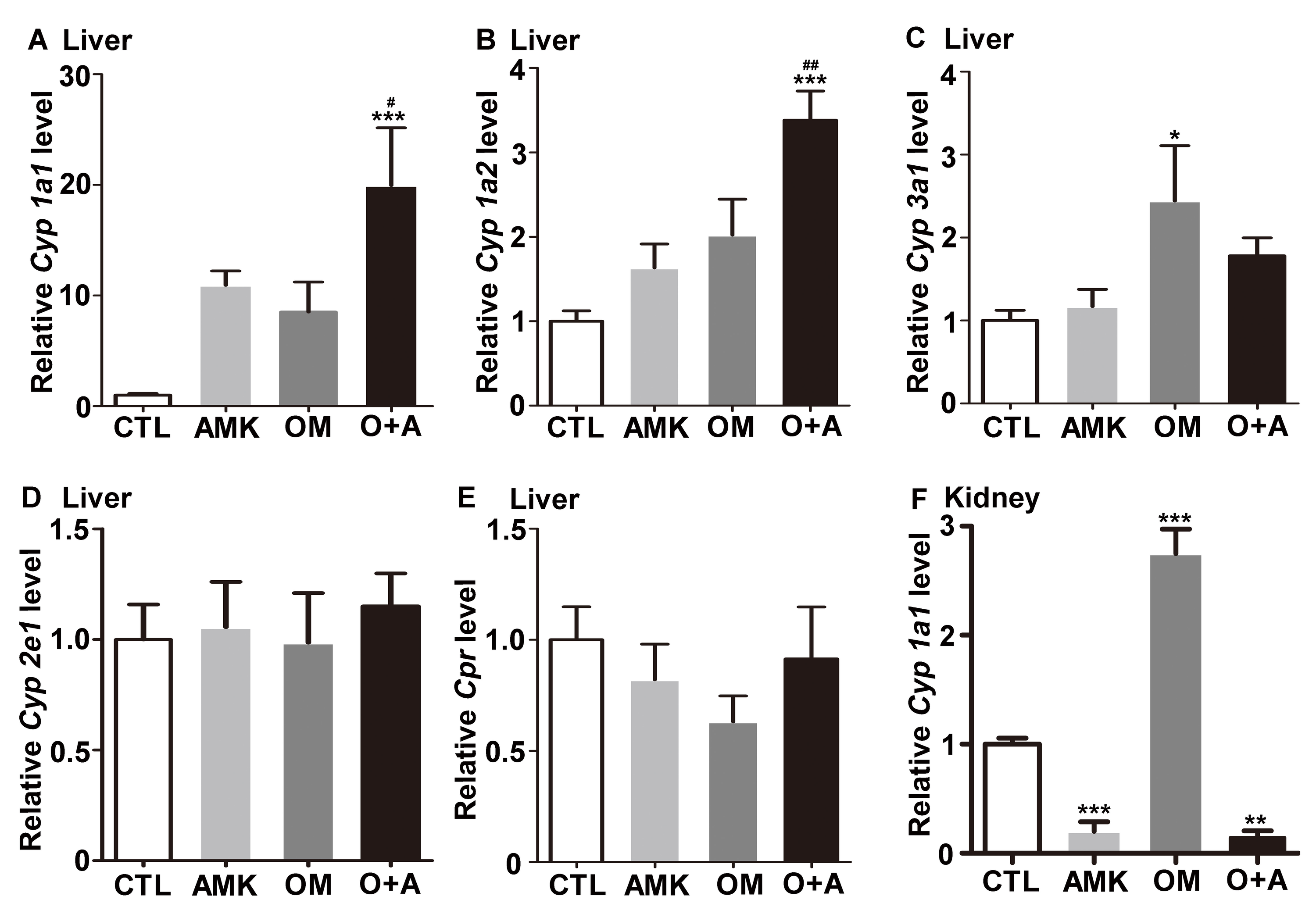

Supplement: S3 Fig — qPCR analysis of hepatic (A) Cyp 1a1, (B) Cyp 1a2, (C) Cyp 3a1, (D) Cyp 2e1, and (E) Cpr and renal (F) Cyp 1a1 mRNA levels in the four rat cohorts. CTL, control; AMK, Aristolochia manshuriensis Kom; OM, omeprazole; O+A, omeprazole and AMK. Data are expressed as mean ± SEM. *P<0.05 vs. CTL cohort; **P<0.01 vs. CTL cohort; ***P<0.001 vs. CTL cohort; # P<0.05 vs. AMK cohort; ## P<0.01 vs. AMK cohort. n = 6. (TIF) [file pone.0164215.s003.tif]
