# Supplementary material for: Omeprazole Alleviates Aristolochia manshuriensis Kom-Induced Acute Nephrotoxicity
Source: PLoS One. 2016 Oct 7;11(10):e0164215. doi: 10.1371/journal.pone.0164215 (PMC5055352; doi:10.1371/journal.pone.0164215)
Supplement: S1 Table — (DOC) [file pone.0164215.s004.doc]

S1 Table. qPCR primer sequence

| species | gene | Primer sequence |
| --- | --- | --- |
| Rat | *Cyp 1a1* | Forward: 5’-TAACTCTTCCCTGGATGCCTTCAA-3’  Reverse: 5’-GTCCCGGATGTGGCCCTTCTCAAA-3’ |
|  | *Cyp 1a1*2 | Forward: 5’-TGTGGACTTCTTTCCGGTCC-3’  Reverse: 5’-GTCCTGGATACTGTTCTTGTTGAAG-3’ |
|  | *Cyp 3a1* | Forward:5’-TCTGTGCAGAAGCATCGAGTG-3’ Reverse:5’-TGGGAGGTGCCTTATTGGG-3’ |
|  | *Cyp 2e1* | Forward:5’-GGTTTTCCCTAAGCATTCTCCG-3’  Reverse: 5’-GGTCTTTTTGAGCTCCTCCACC-3’ |
|  | *Cpr* | Forward: 5’-CTCACGCAGCTTAATGTGGC-3’  Reverse: 5’-GAGCATCCCCGCACACATAG-3’ |
|  | *Ngal* | Forward: 5’-GATGAACTGAAGGAGCGATTC-3’  Reverse: 5’-TCGGTGGGAACAGAGAAAAC-3’ |
|  | *GAPDH* | Forward: 5’-CCTGGAGAAACCTGCCAAGTAT-3’  Reverse: 5’-AGCCCAGGATGCCC TTTAGT-3’ |
| Homo | *CYP 1A1* | Forward: 5’- ACATGCTGACCCTGGGAAAG-3’  Reverse: 5’- GGTGTGGAGCCAATTCGGAT-3’ |
|  | *Actin* | Forward: 5’- GAGCTGCGTGTGGCTCCC-3’  Reverse: 5’- CCAGAGGCGTACAGGGATAGCA-3’ |
